# Comparison of the performance of mental health, drug and alcohol comorbidities based on ICD-10-AM and medical records for predicting 12-month outcomes in trauma patients

**DOI:** 10.1186/s12913-018-3248-x

**Published:** 2018-06-05

**Authors:** Tu Q. Nguyen, Pamela M. Simpson, Sandra C. Braaf, Peter A. Cameron, Rodney Judson, Belinda J. Gabbe

**Affiliations:** 1 Department of Epidemiology and Preventive Medicine, Monash University, Melbourne, Victoria Australia; 2 Emergency and Trauma Centre, The Alfred Hospital, Melbourne, Victoria Australia; 3 Trauma Service, Royal Melbourne Hospital, Melbourne, Victoria Australia; 4 Farr Institute, Swansea University Medical School, Swansea University, Swansea, UK

**Keywords:** Trauma, Mental comorbidities, Post-injury outcomes, Prediction, Validation, Medical record coding

## Abstract

**Background:**

Many outcome studies capture the presence of mental health, drug and alcohol comorbidities from administrative datasets and medical records. How these sources compare as predictors of patient outcomes has not been determined. The purpose of the present study was to compare mental health, drug and alcohol comorbidities based on ICD-10-AM coding and medical record documentation for predicting longer-term outcomes in injured patients.

**Methods:**

A random sample of patients (*n* = 500) captured by the Victorian State Trauma Registry was selected for the study. Retrospective medical record reviews were conducted to collect data about documented mental health, drug and alcohol comorbidities while ICD-10-AM codes were obtained from routinely collected hospital data. Outcomes at 12-months post-injury were the Glasgow Outcome Scale – Extended (GOS-E), European Quality of Life Five Dimensions (EQ-5D-3L), and return to work. Linear and logistic regression models, adjusted for age and gender, using medical record derived comorbidity and ICD-10-AM were compared using measures of calibration (Hosmer-Lemeshow statistic) and discrimination (C-statistic and R^2^).

**Results:**

There was no demonstrable difference in predictive performance between the medical record and ICD-10-AM models for predicting the GOS-E, EQ-5D-3L utility sore and EQ-5D-3L mobility, self-care, usual activities and pain/discomfort items. The area under the receiver operating characteristic (AUC) for models using medical record derived comorbidity (AUC 0.68, 95% CI: 0.63, 0.73) was higher than the model using ICD-10-AM data (AUC 0.62, 95% CI: 0.57, 0.67) for predicting the EQ-5D-3L anxiety/depression item. The discrimination of the model for predicting return to work was higher with inclusion of the medical record data (AUC 0.69, 95% CI: 0.63, 0.76) than the ICD-10-AM data (AUC 0.59, 95% CL: 0.52, 0.65).

**Conclusions:**

Mental health, drug and alcohol comorbidity information derived from medical record review was not clearly superior for predicting the majority of the outcomes assessed when compared to ICD-10-AM. While information available in medical records may be more comprehensive than in the ICD-10-AM, there appears to be little difference in the discriminative capacity of comorbidities coded in the two sources.

**Electronic supplementary material:**

The online version of this article (10.1186/s12913-018-3248-x) contains supplementary material, which is available to authorized users.

## Background

Injuries accounted for 11% of the global burden of disease in terms of Years Lived with Disability in 2015 [1]. Risk-adjustment analyses of long-term outcomes after injury have included compensable status, injury mechanism, injury severity, perceived level of social support, socioeconomic position and educational level [2–5]. Mental health, drug and alcohol comorbidities have been cited as factors that impact on recovery and quality of life following injury [6, 7]. However, the importance of these comorbidities may be under-estimated in risk modelling of long-term outcomes [8]. Data sources used in research studies to capture comorbid conditions are ostensibly linked to the research question, patient population and resources available. Consequently, poor comorbidity capture in different data sources has an impact on the resulting comorbidity profile of injured people and the ability to predict patient outcomes after injury.

Although time- and labour-intensive to review, medical records represent a comprehensive source of comorbidity data, due to the detailed clinical information contained within the notes [9]. Alternatively, routinely collected administrative data are commonly relied on in epidemiological research as they are relatively cost-effective and readily available [10]. Findings from previous research suggest that mental health, drug and alcohol comorbidities derived from administrative data has been shown to be a strong predictor of in-hospital mortality [11], hospital-acquired infection [12] and longer hospital length of stay [13]. However, prediction of longer-term recovery outcomes using comorbidities based on administrative data have generated inconsistent results [14–16]. This could be explained by biases in administrative data, such as those associated with collecting data in order to account for different types of patient episodes and services provided, in the case of activity-based funding [17].

Direct comparisons between administrative coded mental health, drug and alcohol diagnoses with other sources of data in injured populations are scarce [18, 19]. The completeness of administrative coding largely relies on the quality of the medical record documentation but little is known about the impact of using the two alternative data sources on long-term outcome modelling. The aim of this study was to compare the contribution of two different sources of mental health, drug and alcohol comorbidity data in predicting 12-month functional, return to work and health-related quality of life outcomes in a major trauma population.

## Methods

### Study design and inclusion criteria

The Victorian State Trauma Registry (VSTR) is a population-based registry which captures information about virtually all major trauma patients across the state of Victoria [20]. The VSTR follows up all adult survivors to discharge after their injury including function, health-related quality of life (HRQoL), return to work and post-discharge mortality. Standardised telephone interviews are conducted by trained interviewers [21]. A random sample of 500 patients registered on the VSTR were selected for the study. Patients were eligible for inclusion in this study if they met each of the following criteria:

i. Aged 18 years or older;
ii. Definitively managed at one of the two adult major trauma services in Victoria;
iii. Survived to 12 months post-injury and 12-month follow-up completed.

### Procedures

In Australia, hospital data are collected in the form of International Statistical Classification of Diseases, Tenth Revision, Australian Modification (ICD-10-AM) codes and apply to all public and private hospitals. In Victoria, each code contains a prefix with “P” representing principal diagnosis requiring treatment during the stay, “A” representing additional diagnoses and “C” representing in-hospital complications. These codes are assigned by the hospital coders as part of routinely collected hospital data and are used for activity-based funding purposes [22].

Data were extracted from the VSTR for patients in the sample including clinical and demographic data and follow-up outcome data collected at 12-months post-injury. This included patients’ compensation status by the no-fault, third party insurers for road traffic injury and work-related injury and the Charlson Comorbidity Index (CCI), a weighting used to assess comorbidity [23]. The ICD-10-AM “P” and “A” prefixed codes, which are provided to the VSTR by the hospitals, were also extracted. Retrospective review of the medical records of the sample of patients was conducted, by the author of this paper in consultation with researchers with clinical and health expertise, at the two adult major trauma services.

To enable comparison with ICD-10-AM codes, all mental health, drug and alcohol comorbidities described in the medical record were captured based on the categories of mental and behavioural disorder (Chapter V) specified by ICD-10-AM. If a mental health, drug or alcohol condition was documented, based on clinical notation in addition to substantiating evidence such as an alcohol withdrawal scale or psychiatrist referral letter for ongoing treatment, this was recorded on a project data collection form in addition to the specified diagnosis. Pre-existing mental health disorders were captured from the medical record based on the following categories: organic mental disorders, schizophrenia, mood disorders, neurotic disorders, behavioural and personality disorders and other specified mental disorders.

A single binary indicator variable were generated to represent the presence of ICD-10-AM coded mental, drug and alcohol use disorders. This indicator variable included the major categories: alcohol use disorders (F10), drug use disorders (F11-F15, F16, F19) and mental disorders (F00-F03, F07, F09, F20-F48, F60-F69, F90-F99, U792, U793).

### Outcome measures

The primary outcome measure recorded on the VSTR used to assess patients’ level of function is the Glasgow Outcome Scale-Extended (GOS-E). The GOS-E classifies patients into eight levels of function from death (GOS-E = 1), upper severe disability (GOS-E = 4) to upper good recovery (GOS-E = 8), indicating a complete return to normal activities of daily life [24]. The three-level European Quality of Life Five Dimensions (EQ-5D-3L) is used to measure of health-related quality of life and consists of five dimensions (mobility, self-care, usual activities, pain/discomfort, and anxiety/depression), with each item having three possible responses: no problems, some problems and extreme problems. The EQ-5D-3L item responses are used to calculate a single utility score using age and gender specific social preference weights [25]. Algorithms were used to convert EQ-5D-3L responses into health utility scores [range: 0 (equivalent to death) to 1 (equivalent to full health)]. The original preference weights for the EQ-5D-3L from the UK population were applied as these are most commonly used [25]. If patients worked or studied prior to injury, they were asked whether they had returned to work or study.

To establish whether the capacity to predict patient outcomes at 12-months post-injury differed between medical record review of drug, alcohol and mental health comorbidities and those coded in ICD-10-AM, comparison of model performance was undertaken. A ‘good recovery’ was defined as a GOS-E score of 7–8 and a score less than seven represented ongoing functional limitations. The EQ-5D-3L item responses were dichotomised as no problems versus some or severe problems.

### Analysis

In order to detect a statistically significant difference between the area under the Receiver Operating Characteristic (AUC) of 0.7 and 0.8 with a 5% one-sided type I error and 80% power, 158 patients was the smallest sample size required for each hospital [26]. We over-sampled patients beyond this minimum sample size and accessed the medical records of 250 patients from each hospital, in order to account for issues with availability of paper medical records and missing volumes which are common in medical record review studies [27]. Binary logistic regression models were fitted for the categorical outcomes functional recovery, return to work, and the EQ-5D-3L items of mobility, self-care, usual activities, pain/discomfort and anxiety/depression. A linear regression model was fitted for the continuous outcome EQ-5D-3L utility score. Age was categorised for inclusion in the models as it was not linearly related to the log odds of the outcomes of interest for the logistic models in its continuous form.

The following models were fitted for each the outcomes measured:

i) Age and gender;
ii) Medical record coded mental health, drug and alcohol comorbidities, adjusted for age and gender
iii) ICD-10-AM coded mental health, drug and alcohol comorbidities, adjusted for age and gender

Discrimination was evaluated using the C-statistic, or area under the receiver operating characteristic curve (AUC), which ranges from zero to one, with a value of 0.5 representing a model with no predictive power and a value of 1 indicating a model with perfect predictive power. A value between 0.7 and 0.8 is considered acceptable predictive performance, while values greater than 0.8 demonstrate excellent predictive performance [28]. To investigate model fit, likelihood ratio (LR) tests were used. A higher LR statistic and significant *p*-value supports the hypothesis that including the comorbidity data was a significant improvement in model fit over the model of age and gender alone [29]. Hosmer-Lemeshow (H-L) statistics were used to compare model calibration of logistic regression models [28]. A linear regression model was fitted for the EQ-5D-3L utility scores outcome and the coefficient of determination (R^2^) of the models used to assess predictive performance. A *p*-value < 0.05 was considered significant. All analyses were conducted using Stata 13.0 (StataCorp, College Station, TX).

## Results

The clinical and demographic characteristics of the study sample are shown in Table 1. There was an even distribution of patients among the age groups and most patients were male. The median (IQR) injury severity score was 17 (14–22), and these figures are representative of major trauma patients in Victoria [30]. Most patients did not have a Charlson Comorbidity Index condition recorded and did not claim compensation for their injuries (Table 1).

**Table 1:** Demographic and clinical characteristics of patients within the sample (*n* = 500)

Variable	Total n (%)
Age (years)	
18–24	63 (12.6)
25–34	92 (18.4)
35–44	98 (19.6)
45–54	91 (18.2)
55–64	72 (14.4)
≥ 65	84 (16.8)
Gender	
Male	374 (74.8)
Female	126 (25.2)
Charlson Comorbidity Index weighting	
0	351 (70.2)
1	115 (23.0)
≥ 2	34 (6.8)
Compensation status^a^	
Compensable	180 (36.1)
Non-compensable	318 (63.9)

^a^Compensation status not available for *n* = 2

Based on the GOS-E measure, 41% (*n* = 205) of patients in the sample reported a good recovery at 12-months post-injury. According to the H-L statistic, the models predicting functional recovery demonstrated acceptable calibration. Inclusion of the mental health, drug and alcohol comorbidity data, from either the medical record or ICD-10-AM, improved the model fit but none of the models showed acceptable discrimination (Table 2) (Additional file 1).

**Table 2:** Discrimination and calibration statistics for predicting GOS-E good recovery and return to work

	Good recovery (GOS-E 7–8)			
Model	N	AUC (95% CI)	H-L statistic (*p* value)	LR-test (*p*-value)
Age and gender	500	0.57 (0.52, 0.63)	2.3 (0.89)	–
Age, gender and medical record data	500	0.62 (0.57, 0.67)	7.1 (0.52)	11.7 (< 0.01)^a^
Age, gender, ICD-10-AM	500	0.58 (0.53, 0.63)	3.3 (0.91)	0.7 (0.42) ^a^
	Return to work			
	N	AUC (95% CI)	H-L statistic (*p* value)	LR-test (*p*-value)
Age and gender	348	0.55 (0.49, 0.62)	4.3 (0.75)	–
Age, gender and medical record data	348	0.69 (0.63, 0.76)	7.5 (0.48)	38.1 (< 0.01) ^a^
Age, gender, ICD-10-AM	348	0.59 (0.52, 0.65)	3.6 (0.89)	5.9 (0.01) ^a^

^a^Compared to age and gender model

Three hundred and thirty eight (68%) patients reported that they were working or studying prior to injury. Of those who were working/studying prior to injury, 71% (*n* = 240) had returned to work or study at 12-months post-injury. The models predicting return to work all showed less than acceptable discrimination but demonstrated acceptable calibration (Table 2). The addition of the medical record data, but not the ICD-10-AM coded data, improved discrimination over age and gender.

The EQ-5D-3L was complete for 98% of patients at 12 months. The mean (SD) EQ-5D-3L utility score in the sample was 0.75 (0.29). The prevalence of problems was highest for the mobility and anxiety or depression items (Table 3). Overall, the prevalence of problems was lowest for the self-care item.

**Table 3:** EQ-5D-3L responses at 12-months post-injury

EQ-5D-3L dimension	n (%)
Mobility^a^	
No problems	337 (68.4)
Some or severe problems	156 (31.6)
Self-care ^a^	
No problems	398 (80.8)
Some or severe problems	95 (19.3)
Usual activities^b^	
No problems	263 (53.5)
Some or severe problems	229 (46.5)
Pain or discomfort ^a^	
No problems	278 (56.4)
Some or severe problems	215 (43.6)
Anxiety or depression ^a^	
No problems	316 (64.1)
Some or severe problems	177 (35.9)

^a^Missing data (*n* = 7) for mobility, self-care, pain/discomfort and anxiety/depression

^b^Missing data (*n* = 8) for usual activities

The models predicting problems on each of the dimensions of the EQ-5D-3L showed less than acceptable discrimination (Table 4). The capacity to discriminate between patients with and without problems in the mobility, self-care, usual activities, pain/discomfort items and utility score was similar for the ICD-10-AM and medical record models except for patients with anxiety/depression problems. For predicting the EQ-5D-3L utility score, the highest R^2^ was observed for the medical record model. Both models were an improvement over the age and gender model, but addition of the ICD-10-AM coded conditions was only a marginal improvement over age and gender (Table 4). Calibration was acceptable according to the H-L statistic for predicting all of the EQ-5D-3L items.

**Table 4:** Discrimination and calibration statistics for predicting EQ-5D-3L dimensions and utility score

	EQ-5D Mobility			
Model	N	AUC (95% CI)	H-L statistic (*p*-value)	LR-test (*p*-value)
Age and gender	493	0.56 (0.51, 0.62)	0.6 (0.99)	–
Age, gender and medical record data	493	0.58 (0.53, 0.63)	2.3 (0.97)	4.3 (0.04) ^a^
Age, gender, ICD-10-AM	493	0.56 (0.51, 0.62)	3.7 (0.81)	0.2 (0.64) ^a^
	EQ-5D Self-care			
	N	AUC (95% CI)	H-L statistic (*p*-value)	LR-test (*p*-value)
Age and gender	493	0.58 (0.51, 0.64)	3.6 (0.73)	–
Age, gender and medical record data	493	0.60 (0.53, 0.66)	4.5 (0.80)	4.6 (0.03) ^a^
Age, gender, ICD-10-AM	493	0.57 (0.51, 0.64)	6.8 (0.45)	0.4 (0.54) ^a^
	EQ-5D Usual activities			
	N	AUC (95% CI)	H-L statistic (*p*-value)	LR-test (*p*-value)
Age and gender	492	0.57 (0.52, 0.62)	1.8 (0.94)	–
Age, gender and medical record data	492	0.59 (0.54, 0.64)	5.1 (0.75)	6.0 (0.01) ^a^
Age, gender, ICD-10-AM	492	0.57 (0.52, 0.62)	6.2 (0.63)	0.1 (0.72) ^a^
	EQ-5D Pain or discomfort			
	N	AUC (95% CI)	H-L statistic (*p*-value)	LR-test (*p*-value)
Age and gender	493	0.58 (0.53, 0.63)	3.2 (0.78)	–
Age, gender and medical record data	493	0.58 (0.53, 0.63)	8.2 (0.41)	2.7 (0.10) ^a^
Age, gender, ICD-10-AM	493	0.58 (0.53, 0.63)	5.6 (0.58)	0.0 (0.92) ^a^
	EQ-5D Anxiety or depression			
	N	AUC (95% CI)	H-L statistic (*p*-value)	LR-test (*p*-value)
Age and gender	493	0.55 (0.50, 0.60)	1.7 (0.94)	–
Age, gender and medical record data	493	0.68 (0.63, 0.73)	9.9 (0.28)	42.1 (< 0.01) ^a^
Age, gender, ICD-10-AM	493	0.62 (0.57, 0.67)	6.7 (0.57)	10.9 (0.01) ^a^
	EQ-5D-3L utility score			
	N	R^2^	LR-test (*p*-value)	
Age and gender	492	0.01	–	
Age, gender and medical record data	492	0.06	22.1 (< 0.01)^a^	
Age, gender, ICD-10-AM	492	0.02	4.0 (0.04) ^a^	

^a^Compared to age and gender model

## Discussion

Comorbidity information available in different data sources have their own advantages and limitations, which can affect the validity of studies relying on such data [31]. It has been acknowledged that routinely collected administrative data sub-optimally represent a patient’s condition and the totality of their comorbid illnesses, particularly for mental health, drug and alcohol comorbidities which are historically difficult to characterise, classify and diagnose [32]. Previous analyses of administrative coding in routinely collected hospital data compared with medical records have revealed the prevalence of comorbid conditions are frequently under-estimated [33–35]. Observable discrepancies between the data sources exist [36], and are driven largely by coding rules, activity-based funding, variability in coder interpretation and inconsistent or unclear charting [17, 37]. We sought to compare the statistical performance of two commonly used data sources: medical record documentation and ICD-10-AM administrative data. Mental health, drug and alcohol comorbidities were shown to be important for predicting long-term outcomes, as the addition of the comorbidity data fit the data better than age and gender alone. Overall, the addition of comorbidities obtained from medical record review did not provide improvement in discrimination over age and gender, which was comparable with the ICD-10-AM coded comorbidities, for most of the outcomes assessed.

Mental health, drug and alcohol comorbidities are associated with delays in recovery and disability after injury, but the majority of the evidence predicting these outcomes have relied on self-report or clinical interview to assess comorbidity [38–40]. Pre-injury mental health diagnoses derived from the medical record has been associated with poorer function in previous studies [41, 42], whereas ascertaining pre-existing mental health history based on administrative data coding has yielded differing results [16, 43]. Despite the lower prevalence of comorbidities coded in ICD-10-AM than in the medical record, the findings of this study indicate the capacity to predict functional recovery was largely comparable with the medical record. This suggests that key mental health, drug and alcohol comorbidities, which are predictors and have significant impacts on long-term function after injury, are not comprehensively captured in the medical record at the outset. Notably, some comorbidities within the medical record could have been inadvertently excluded from the review due to missing documentation or illegible handwriting. As a result, mental health, drug and alcohol comorbidities obtained from the medical record may not be reliably predictive of long-term function. Further research is needed to compare the predictive performance of medical record data with self-report and clinical interview to assess these comorbidities for long-term outcome modelling.

Mental health, drug and alcohol comorbidity data derived from medical records have been used previously for risk-adjustment in studies of return to work [42, 44]. Mental health, drug and alcohol comorbidities coded in ICD-10-AM may not be as reliable or be able to perform to the same capacity as medical record abstracted conditions for predicting return to work. This is difficult to assess, as few existing studies have evaluated the association between ICD-10 coded mental health comorbidities and returning to work after injury. A previous study of major trauma patients using ICD-10-AM coded data reported with no significant association with returning to work [15], but this may have been attributed to limited statistical power. While the medical record may provide a high degree of detail about mental health factors that are associated with lower odds of returning to work following severe injury, this information may be incomplete when coded in the ICD-10-AM.

Survivors of traumatic injuries often experience deficits in perceived health status, including pain or discomfort, and difficulty with usual activities and mobility [45]. Aside from anxiety or depression, there were no differences in the discriminative ability of the ICD-10-AM and medical record data for predicting the remaining EQ-5D-3L dimensions or utility score. Neither the medical record documentation nor the ICD-10-AM data were able to differentiate between groups at risk of ongoing problems with mobility, self-care, usual activities and pain/discomfort with any great accuracy. An explanation for this finding may be that self-reported measures of comorbidity may be a better predictor of the EQ-5D-3L outcomes whereas indirect comorbidity measures may not be optimal for predicting these health status outcomes [46]. This suggests the importance of other factors not explained by mental health, drug and alcohol comorbidities including personal and environmental factors for predicting HRQoL following injury. Other factors that have been shown to be important in the literature include compensable status, education, and social circumstances [47–49].

The strengths of the study were the use of long-term outcomes data at 12-months post-injury and the random sample of a population-based cohort, which was representative of major trauma patients in Victoria. Although patients from each hospital were over-sampled in order to mitigate potential issues with missing charts, only seven patient records (3%) of the total sample could not be located, and these patients were replaced from the VSTR. A limitation of the study includes the extent to which these findings may be extrapolated to other patients. This research relied on ICD-10-AM recorded mental health, drug and alcohol diagnoses which were originally collected primarily for activity-based funding. The findings may not be applicable to other jurisdictions, which vary in coding practices and funding systems. This aim of the study was not to develop the best prediction model for long-term outcomes. The selection of variables to identify the ‘optimal’ risk prediction model is based on trying to choose the best predictors of the outcome [50], and the results cannot be interpreted for this purpose without careful selection and further examination of other variables.

## Conclusion

While the prevalence of mental health, drug and alcohol comorbidities is lower in ICD-10-AM than medical records, data derived from medical record review was not clearly superior for predicting the majority of the outcomes assessed. There was no demonstrable difference in discriminative capacity between the medical record and ICD-10-AM models for predicting the GOS-E, EQ-5D-3L utility sore and EQ-5D-3L mobility, self-care, usual activities and pain/discomfort items. Further research is needed to compare the contribution of medical record documented mental health, drug and alcohol comorbidities with alternative data sources.

## Additional file

Additional file 1: **Figure S1.** Receiver operating characteristic curves for the models incorporating medical record and ICD-10 data predicting a good recovery (GOS-E 7–8), **Figure S2.** Receiver operating characteristic curves for the models incorporating medical record and ICD-10 data predicting return to work, **Figure S3.** Receiver operating characteristic curves for models incorporating medical record and ICD-10 data predicting EQ-5D-3L problems with ongoing mobility, **Figure S4.** Receiver operating characteristic curves for models incorporating medical record and ICD-10 data predicting EQ-5D-3L problems with ongoing self-care, **Figure S5.** Receiver operating characteristic curves for models incorporating medical record and ICD-10 data predicting EQ-5D-3L ongoing problems with usual activities, **Figure S6.** Receiver operating characteristic curves for models incorporating medical record and ICD-10 data predicting EQ-5D-3L problems with ongoing pain or discomfort, **Figure S7.** Receiver operating characteristic curves for models incorporating medical record and ICD-10 data predicting EQ-5D-3L problems with ongoing anxiety or depression (DOCX 11452 kb)

## Abbreviations

ACS: Australian Coding Standards
AUC: Area under the curve
EQ-5D: European Quality of Life Five Dimensions
H-L: Hosmer-Lemeshow
HRQoL: Health-related quality of life
ICD-10-AM: International Classification of Diseases, Tenth Revision, Australian Modification
ROC: Receiver operating characteristic
VSTR: Victorian State Trauma Registry
