# Supplementary material for: Comparison of the performance of mental health, drug and alcohol comorbidities based on ICD-10-AM and medical records for predicting 12-month outcomes in trauma patients
Source: BMC Health Serv Res. 2018 Jun 5;18:408. doi: 10.1186/s12913-018-3248-x (PMC5989374; doi:10.1186/s12913-018-3248-x)
Supplement: Supplementary file 1 — Figure S1. Receiver operating characteristic curves for the models incorporating medical record and ICD-10 data predicting a good recovery (GOS-E 7–8), Figure S2. Receiver operating characteristic curves for the models incorporating medical record and ICD-10 data predicting return to work, Figure S3. Receiver operating characteristic curves for models incorporating medical record and ICD-10 data predicting EQ-5D-3L problems with ongoing mobility, Figure S4. Receiver operating characteristic curves for models incorporating medical record and ICD-10 data predicting EQ-5D-3L problems with ongoing self-care, Figure S5. Receiver operating characteristic curves for models incorporating medical record and ICD-10 data predicting EQ-5D-3L ongoing problems with usual activities, Figure S6. Receiver operating characteristic curves for models incorporating medical record and ICD-10 data predicting EQ-5D-3L problems with ongoing pain or discomfort, Figure S7. Receiver operating characteristic curves for models incorporating medical record and ICD-10 data predicting EQ-5D-3L problems with ongoing anxiety or depression (DOCX 11452 kb) [file 12913_2018_3248_MOESM1_ESM.docx]

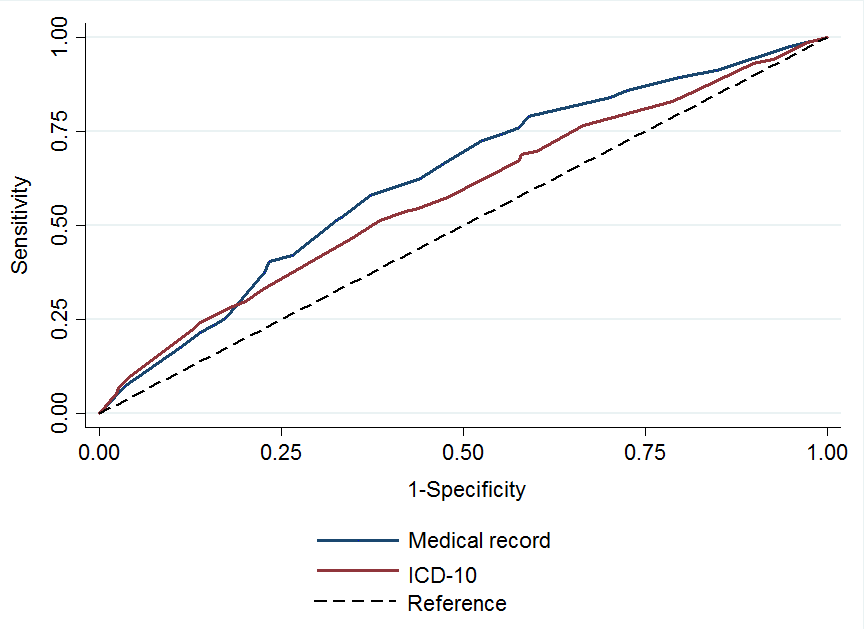


Figure S1: Receiver operating characteristic curves for the models incorporating medical record and ICD-10 data predicting a good recovery (GOS-E 7-8)


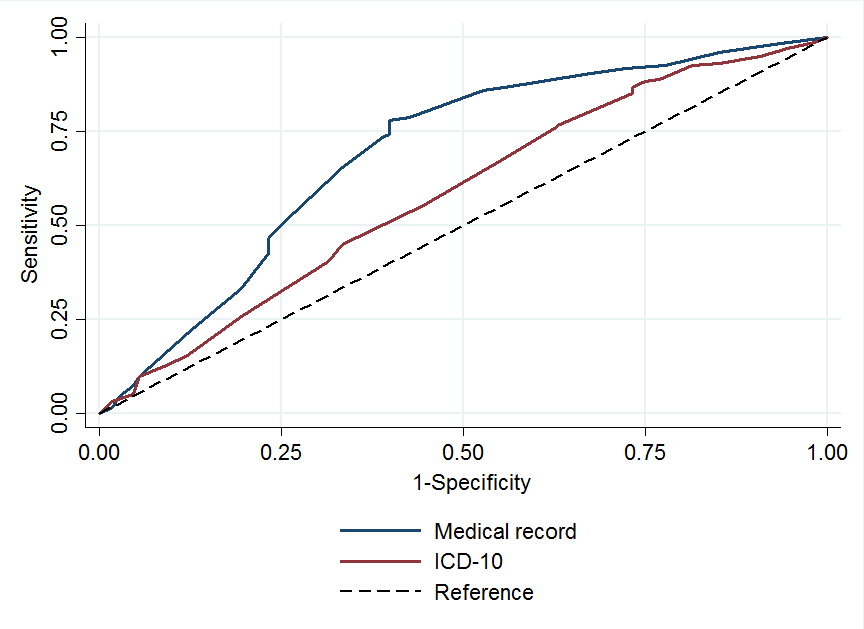


Figure S2: Receiver operating characteristic curves for the models incorporating medical record and ICD-10 data predicting return to work


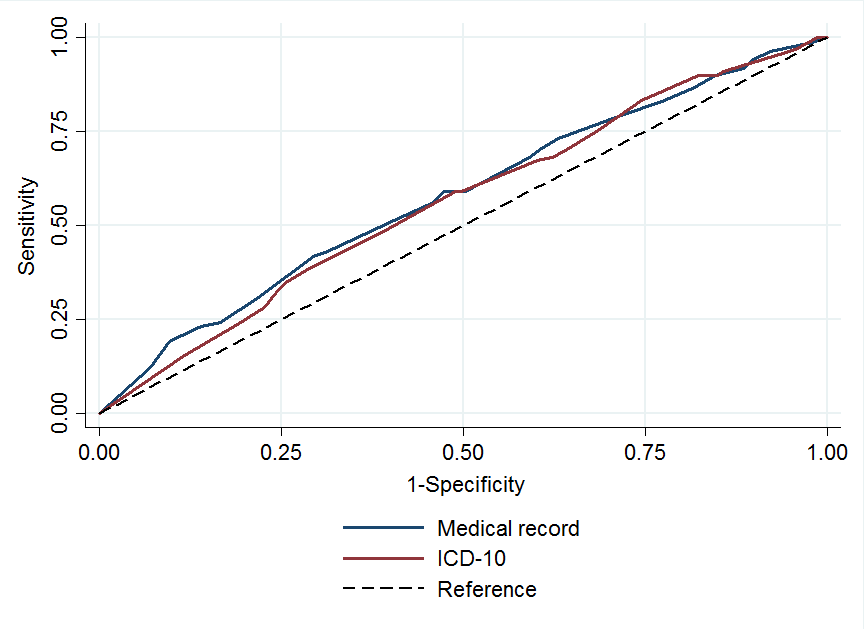


Figure S3: Receiver operating characteristic curves for models incorporating medical record and ICD-10 data predicting EQ-5D-3L problems with ongoing mobility


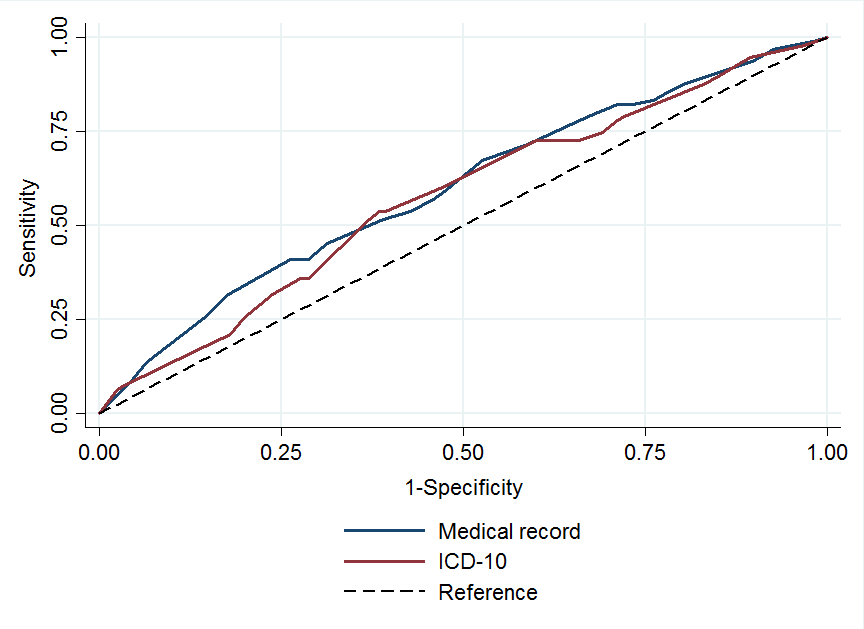


Figure S4: Receiver operating characteristic curves for models incorporating medical record and ICD-10 data predicting EQ-5D-3L problems with ongoing self-care


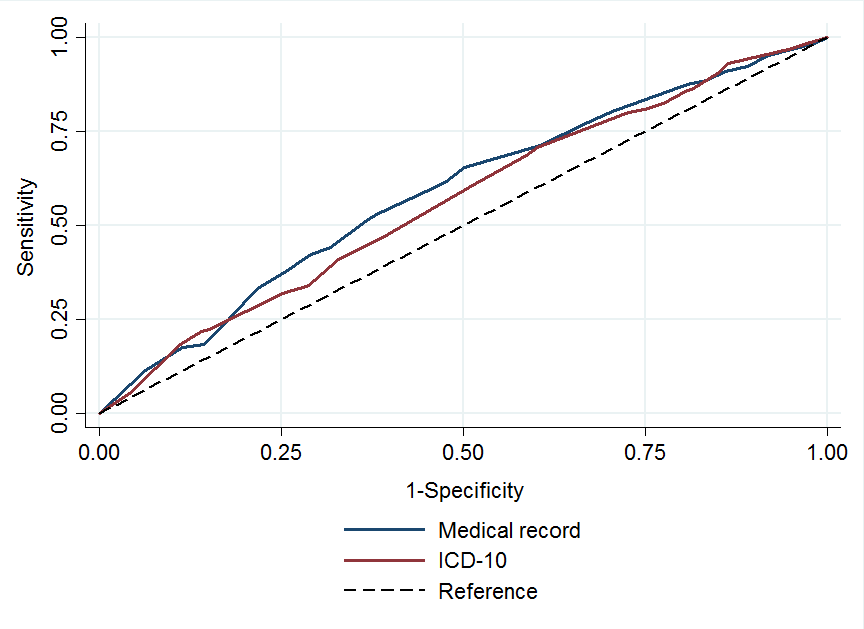


Figure S5: Receiver operating characteristic curves for models incorporating medical record and ICD-10 data predicting EQ-5D-3L ongoing problems with usual activities


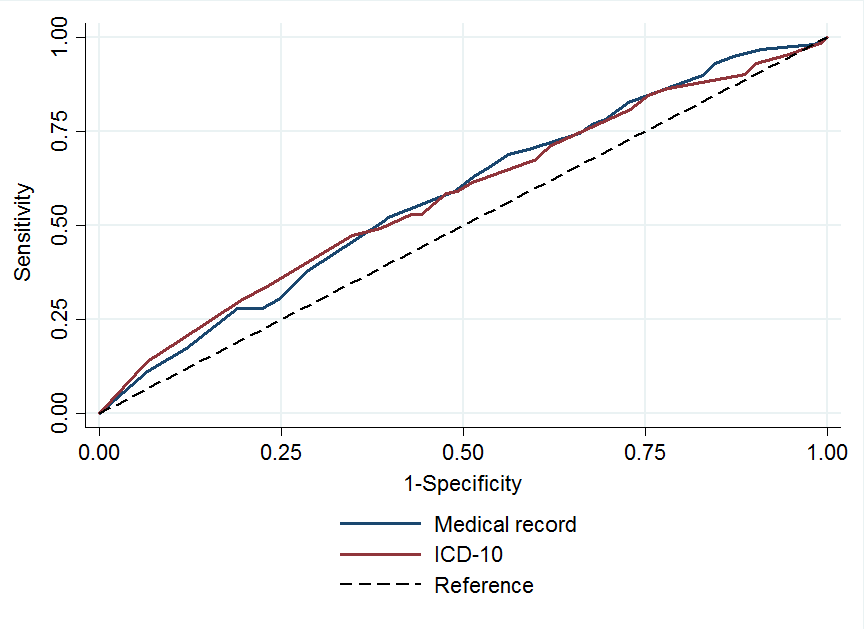


Figure S6: Receiver operating characteristic curves for models incorporating medical record and ICD-10 data predicting EQ-5D-3L problems with ongoing pain or discomfort


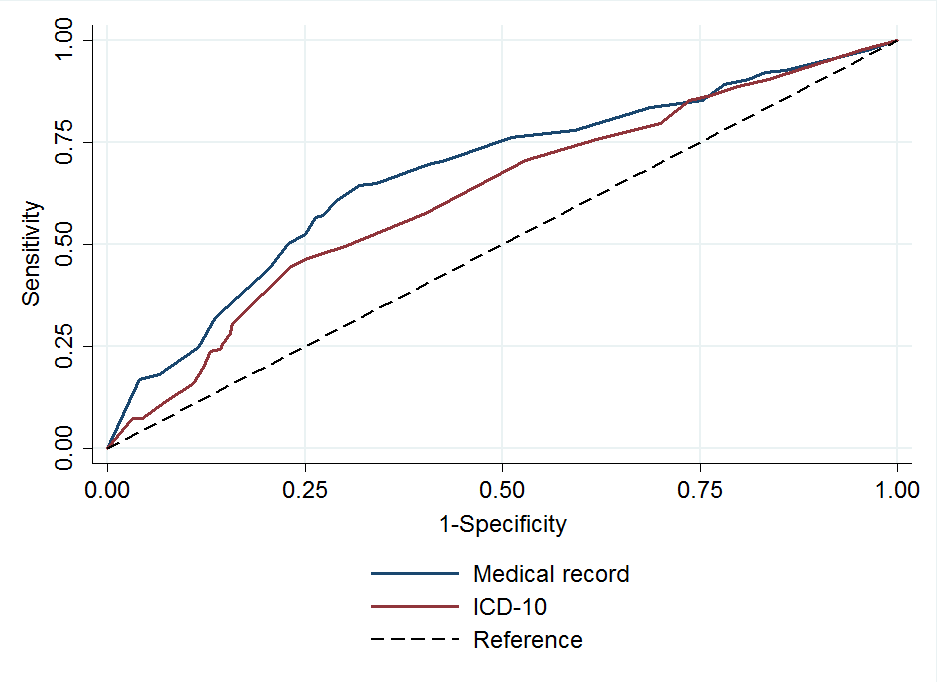


Figure S7: Receiver operating characteristic curves for models incorporating medical record and ICD-10 data predicting EQ-5D-3L problems with ongoing anxiety or depression
